# Global Proteomic Analysis of Sperm and Seminal Plasma From Tropically‐Adapted Rams With Contrasting Parameters of Frozen‐Thawed Semen

**DOI:** 10.1002/mrd.70148

**Published:** 2026-10-04

**Authors:** Wallisson Bruno de Morais Pacheco, Iran Aguila Maciel, Suane Santos Alves dos Santos, Alan Martins de Araújo, Francisca Nágila Matos Almeida, Denise Damasceno Guerreiro, Sheheryar Sheheryar, Lais Lacerda Brasil de Oliveira, Felipe Domingos de Sousa, Mohibullah Shah, Maria Elisabete Amaral Moraes, Carlos Roberto Koscky Paier, Ana Cristina de Oliveira Monteiro, Carminda Sandra Brito Salmito‐Vanderley, Arlindo A. Moura

**Affiliations:** ^1^ Graduate Program in Veterinary Sciences, School of Veterinary Medicine Ceará State University Fortaleza Brazil; ^2^ Graduate Program in Animal Science, Department of Animal Science Federal University of Ceará Fortaleza Brazil; ^3^ School of Veterinary Medicine Ceará State University Fortaleza Brazil; ^4^ Drug Research and Development Center, School of Medicine Federal University of Ceará Fortaleza Brazil; ^5^ Center of Experimental Biology The University of Fortaleza (Unifor) Fortaleza Brazil

**Keywords:** cryopreservation, proteome, rams, seminal plasma, spermatozoa

## Abstract

The aim of this study was to characterize the proteome of sperm and seminal plasma (SP) from rams with high semen freezability (HF) and low semen freezability (LF). Eleven ejaculates were collected from 12 rams, at 3‐day intervals, and cryopreserved (132 semen samples). For proteomic analysis, sperm and SP samples were pooled separately for each ram. Pools 1 and 2 included ejaculates obtained on odd‐numbered and even‐numbered collection days, respectively. This generated 24 sperm and 24 SP pools. Sperm and SP proteins were analyzed by mass spectrometry, and computational biology platforms, used for calculation of differentially abundant proteins (DAPs; *p* < 0.05) in HF (*n* = 5) and LF (*n* = 7) rams. Total sperm motility decreased from 91.87% ± 0.63% in fresh to 65.65% ± 2.58% in frozen‐thawed semen of HF rams; and decreased from 89.47% ± 1.15% to 50.05% ± 2.33% in LF rams. Reduction in total sperm motility after cryopreservation was lower in HF (26.22% ± 2.39%) compared to LF rams (37.70% ± 2.23%; *p* = 0.006). Sperm progressive motility decreased less in HF rams (from 87.20% ± 0.50% to 58.12% ± 2.82%) than in LF rams (from 85.54% ± 1.41% to 44.00 ± 2.13; *p* = 0.006). Rams with the HF phenotype had better plasma membrane (39.00% ± 2.79% vs. 15.14% ± 0.92%) and acrosome integrity (37.54% ± 2.72% vs. 15.40% ± 1.92%; *p* < 0.001) than LF rams. Sperm motility was similar in fresh semen of HF and LF rams. There were 1599 and 1677 sperm proteins identified in HF and LF rams, with 73 and 76 DAPs, respectively. SP had 1127 proteins, with 25 and 45 DAPs in HF and LF rams. This is the greatest coverage ever published about the ram SP proteome. Sperm proteins with greater abundance in HF rams were extracellular and immune‐related proteins, antiproteases, chaperones and structural regulators, contributing to cytoskeletal integrity, apoptotic resistance and maintenance of proteostasis. Sperm proteins with greater abundance in LF rams were mainly mitochondrial subunits, axonemal components, and bioenergetic enzymes, indicating greater reliance on oxidative processes. Major components of HF SP participate in sperm protection, stabilization of membrane lipids, calcium fluxes, epididymal maturation and ATP availability. The major LF SP proteome was associated with cellular stress, inflammatory responses, oxidative damage and ATP production. Thus, the nature of sperm and SP proteome atlas potentially defines ram sperm cryoresilience.

## 1 Introduction

Cryopreservation of semen is a biotechnological tool with remarkable applications for germplasm cryobanks and genetic selection of commercial herds. However, its efficiency is limited by factors such as individual variability among individuals and the cellular damage caused by freezing and thawing, especially in the plasma and acrosomal membranes (Khan et al. 2021). These damages compromise viability, motility, and sperm fertilizing capacity of frozen‐thawed sperm (Yánez‐Ortiz et al. 2022).

During cryopreservation, spermatozoa are exposed to physical and chemical stresses that compromise their functionality. Osmotic stress and oxidative stress emerge as central factors, resulting from rapid temperature changes and contact with cryoprotectants, which affect lipid fluidity and protein conformation (Peris‐Frau et al. 2020). Cryopreservation of ram semen induces mitochondrial dysfunction and a significant increase in oxidative stress, resulting in decreased motility, impairment of structural integrity, loss of mitochondrial potential, and ATP reduction, in addition to the activation of apoptotic markers and cellular damage (Vozaf et al. 2022; Zhang et al. 2024; X. Sun et al. 2024). Similar findings have also been reported in cattle, reinforcing mitochondrial vulnerability as a determinant of cryotolerance (Gacem et al. 2023; Tamargo et al. 2024).

Structural and functional alterations in sperm compromise cellular performance after cryopreservation, resulting in distinct phenotypes among males. Proteomic studies have contributed to understanding these processes, identifying proteins and pathways associated with sperm competence. Differences in the abundance of sperm proteins related to energy metabolism, motility, and membrane integrity were observed among rams with contrasting fertility status, indicating molecular mechanisms that may also be involved in sperm quality (Hitit et al. 2021). Cryopreservation remodels the sperm proteome, as proteins associated with motility, acrosomal function, and control of reactive oxygen species are differentially abundant between fresh and frozen‐thawed ram semen (Lv et al. 2020). Similarly, individual variation in semen freezing capacity is linked to seminal plasma proteins. Proteins such as calgranulin A, calgranulin B, S100 protein A14, S100 protein A16, hypoxia‐upregulated protein 1, peroxiredoxin‐1, and heat shock protein 90 beta member 1 were reported by Ren et al. (2023) as being more abundant in the seminal plasma of high‐freezability (HF) rams. Such molecules relate to antioxidant mechanisms, energy homeostasis, and protection against apoptosis. Furthermore, freezing and thawing cause significant changes in the sperm proteome of rams, with reductions and increases in several proteins associated with metabolic processes and stress responses (Jia et al. 2022). Recent studies have shown that changes in sperm protein phosphorylation patterns are associated with loss of motility and structural dysfunction after thawing. Quantitative phosphoproteomic analyses revealed modifications in ram sperm proteins including A‐kinase anchoring protein 3, A‐kinase anchoring protein 4, calcium‐binding tyrosine phosphorylation‐regulated protein, fibrous sheath‐interacting protein 2, glycogen synthase kinase 3 alpha/beta, glucose‐6‐phosphate isomerase, and outer dense fiber protein 1/2, all of which are involved in regulating energy production and fibrous sheath organization, contributing to the elucidation of the molecular mechanisms associated with reduced post‐thawing motility (Zang et al. 2024). In the context of cryopreservation, an integrated transcriptome–proteome approach identified the membrane protein Fc gamma receptor Ia (FCGR1A) as an important regulator of the fertilizing capacity of ram spermatozoa. FCGR1A expression was significantly reduced in sperm cells after freezing, both at the mRNA and protein levels. Experimental blockade of FCGR1A activity with antibodies resulted in significant decreases in sperm viability, motility, and in vitro embryonic cleavage rate, highlighting its direct functional role in the reproductive process in sheep (Bai et al. 2023). Therefore, variations in both sperm and seminal plasma proteome may explain, at least in part, the individual variability in semen cryotolerance. The present study was conducted to evaluate the associations of semen freezability with sperm and seminal plasma proteome of tropically‐adapted rams.

## 2 Materials and Methods

### 2.1 Experimental Design

In the present study, 11 ejaculates were collected from 12 hairy rams, at 3‐day intervals. All semen samples (132 total) were cryopreserved and sperm kinetics, viability, sperm membrane and acrosome integrity, DNA integrity, and morphology were evaluated in fresh and frozen‐thawed semen. Rams were categorized into high semen freezability (HF) (*n* = 5) and low semen freezability (LF) (*n* = 7) groups based on sperm motility after freezing and thawing. Sperm and seminal plasma samples were subjected to mass spectrometry and computational biology analyses, defining proteins with different abundances in the rams with HF and LF phenotypes.

### 2.2 Location and Semen Collections

The experiment was conducted at the Sheep and Goat Unit of the Federal University of Ceará, in Fortaleza, Ceará (3.7427° S; 38.5785° W). Twelve healthy and reproductively sound hair rams, with an average body weight of 50.8 ± 2.3 kg (2–6 years), were housed in pens and fed Tifton (*Cynodon* spp.) hay and concentrate composed of 60% corn and 40% soybean meal, to provide 18% crude protein according to the recommendations of the BR‐Caprinos & Ovinos (2024). Water and mineral salt were supplied ad libitum, and each animal received a daily allowance of 1% of its body weight in concentrate, divided into two equal meals.

### 2.3 Semen Collection and Assessment of Fresh and Frozen‐Thawed Sperm Kinetics

Semen was collected from rams exposed to an estrous ewe, using an artificial vagina pre‐warmed to 39°C (IMV Technologies, France). Immediately after collection, fresh samples were maintained in a 37°C water bath. An aliquot of 40 µL was diluted in 2 mL of Optidux extender warmed at 39°C (Reprodux, São Paulo, Brazil) to assess sperm kinetics using a portable computer‐assisted sperm analysis equipment (AndroScope, Minitube, Germany). The following parameters were evaluated: total motility (TM), progressive motility (PM), curvilinear velocity (VCL), straight‐line velocity (VSL), average path velocity, curvilinear distance, straight‐line distance, average path distance, amplitude of lateral head displacement (ALH), beat‐cross frequency (BCF), head angle, linearity, and straightness.

### 2.4 Semen Cryopreservation

Immediately after collection, semen was diluted in Optidux extender (Reprodux, Brazil) supplemented with OptiXcell 2 (IMV Technologies, France), according to the manufacturer's instructions, and loaded into 0.25‐mL straws (IMV Technologies, France) at a concentration of 150 × 10^6^ motile spermatozoa/mL. The straws were sealed with polyvinyl alcohol and properly identified. Next, the straws were processed in an automated semen freezer (CRYOGEN HSE 750 Portable V4.0, NeoVet, Minas Gerais, Brazil) according to a controlled cryopreservation curve. The protocol was initiated with a positive ramp at 37°C, followed by cooling at a rate of –0.3°C/min until 4°C, where samples were maintained for 4 h. Subsequently, a negative ramp was applied with a cooling rate of –10°C/min to –20°C, followed by –20°C/min to –110°C. At this stage, the straws were placed in pre‐cooled racks and immersed directly into a cryogenic tank containing liquid nitrogen. The controlled freezing protocol of ram semen was standardized by our team during the pre‐experimental phase, using information available in the literature (Saha et al. 2022).

### 2.5 Sperm Viability and Morphology in Fresh and Frozen‐Thawed Semen

An aliquot of fresh semen maintained in a water bath at 37°C was mixed (1:2, vol/vol) with eosin‐nigrosin stain (Reprodux, São Paulo, Brazil) for the assessment of sperm viability. The mixture was used to prepare smears on glass slides by placing a drop near the edge of the slide and extending it with a second slide to form a thin smear, which was allowed to air‐dry. For frozen‐thawed semen, straws were thawed in a water bath at 37°C for 30 s and processed using the same staining and smear preparation described for fresh semen. At least 200 cells per slide were analyzed and categorized as live or dead, depending on dye penetration (Rocha et al. 2015; Rego et al. 2016). For the analysis of sperm morphology in both fresh and frozen‐thawed semen, cells were fixed in formalin‐citrate (1:100) and subsequently stained on slides with rose bengal (Reprodux, São Paulo, Brazil), with smears were also prepared. According to the recommendations of the Brazilian College of Animal Reproduction (Colégio Brasileiro de Reprodução Animal 2013), at least 200 sperm cells per slide were evaluated for the presence or absence of major and minor morphological defects (Rocha et al. 2015).

### 2.6 Sperm Chromatin Integrity in Fresh Semen

Assessment of sperm chromatin integrity in fresh semen was carried out by adding 1 µL of raw semen to microtubes containing 1 mL of PBS buffer. Samples were processed using the sperm chromatin dispersion technique, as described by Viana Neto et al. (2024). For each sample, a slide was prepared with 10 agarose spots (5 mg/mL in Milli‐Q water), where 2 µL of a mixture containing 50 µL of low‐melting‐point agarose (6.5 mg/mL in Milli‐Q water) and 25 µL of semen diluted in PBS were applied. This procedure was repeated for all samples. Twenty sperm cells were evaluated per spot, totaling at least 200 cells per sample. DNA fragmentation was determined based on the presence or absence of chromatin dispersion halos around the sperm head: cells with intact DNA exhibited large halos, whereas cells with fragmented DNA showed small or absent halos.

### 2.7 Categorization of Rams Into HF and LF Phenotypes

Eight months after cryopreservation, two straws from each of the 132 ejaculates were thawed in 37°C water bath for 30 s. After thawing, semen samples were subjected to the analyses of kinetics, morphology, viability, and DNA integrity as described for fresh semen. For each animal, the reduction in total sperm motility (TMR) was calculated as the difference between TM in fresh and frozen‐thawed semen, using the average of two straws per ejaculate. Rams with TMR values below the overall average were classified as having HF, whereas those with TMR values above the average were defined as LF. Values of TMR were tested for normality and homogeneity of variance using the Shapiro–Wilk and Levene tests, respectively. Differences (*p* < 0.05) between HF and LF rams were confirmed using the *t*‐test (R Studio, version 2025.05.1 + 513). The other parameters were statistically compared between HF and LF rams as well.

### 2.8 Sperm Plasma and Acrosomal Membrane Integrity in Frozen‐Thawed Semen

The integrity of sperm membranes in frozen‐thawed semen was evaluated using PI probes (Propidium Iodide, P4170, Sigma–Aldrich, St. Louis, MO, USA) and CFDA (Carboxyfluorescein Diacetate, 21879, Sigma–Aldrich, St. Louis, MO, USA), according to a protocol adapted from Rego et al. (2016). For the assay, 50 µL of thawed semen was diluted in 150 µL of TRIS solution previously warmed to 37°C, followed by incubation with 20 µL of PI and 5 µL of CFDA for 10 min in the absence of light. After incubation, fluorescence emission was recorded under excitation at 492 nm (blue laser, CFDA) and 535 nm (green laser, PI) using epifluorescence microscopy (LMS 710; Zeiss, Germany). Cells were classified according to membrane function, with at least 200 spermatozoa per sample being counted. Cells exhibiting green fluorescence were classified as having intact and functional membranes, whereas those with red fluorescence were classified as having membrane damage (Snoeck et al. 2023).

### 2.9 Mitochondrial Membrane Potential in Frozen‐Thawed Semen

Mitochondrial membrane potential was assessed using the JC‐1 probe (T4069, Sigma–Aldrich, St. Louis, MO, USA). For each sample, 12 µL of JC‐1 was added to microtubes containing 150 µL of TRIS buffer and 50 µL of semen, followed by incubation in the dark for 10 min. Fluorescence was recorded under excitation at 488 nm (blue laser) using epifluorescence microscopy (LMS 710; Zeiss, Germany). At least 200 cells per sample were evaluated. Cells exhibiting bright red‐orange fluorescence with a “torch‐like” appearance were classified as having high mitochondrial membrane potential, whereas cells showing green or low fluorescence were classified as having low mitochondrial membrane potential (Martinez‐Pastor et al. 2004).

### 2.10 Acrosome Reaction in Frozen‐Thawed Semen

The FITC‐PNA probe (FITC conjugate, L7381, Sigma–Aldrich, St. Louis, MO, USA) was used to identify spermatozoa with reacted acrosome. For each thawed sample, a wet smear (5 µL) was prepared on a glass slide with a coverslip and allowed to air‐dry for 5 min. Slides were then stored in a closed box at 4°C for a minimum of 24 h and up to 2 weeks. A working solution was prepared by mixing 20 µL of FITC‐PNA with 480 µL of PBS (10 µg/mL), and 30 µL of this solution was applied to each refrigerated slide, covering the surface by gentle homogenization. Slides were further incubated at 4°C for 20 min in a dark, humidified container. After incubation, samples were rinsed in 50 mL of PBS and evaluated under excitation at 495 nm (blue laser) using epifluorescence microscopy (LMS 710; Zeiss, Germany). At least 200 cells per sample were observed: spermatozoa showing bright green acrosomal fluorescence were classified as having an intact acrosome, whereas those lacking this fluorescence were classified as acrosome‐reacted (Warr et al. 2025). Data related to the percentage of sperm with plasma membrane integrity, mitochondrial membrane potential, acrosome reaction, and chromatin integrity were tested for normality and homogeneity of variances using the Shapiro–Wilk and Levene tests, respectively. Comparisons between HF and LF rams with regard to each variable were performed using the Student's *t*‐test (*p* < 0.05). Statistical analyses were performed in RStudio (version 2025.05) using R software.

### 2.11 General Sample Preparation for Proteomic Analyses

A fraction of fresh semen (approximately 500 µL) not used for cryopreservation was treated with protease inhibitor (P8340, Sigma–Aldrich, St. Louis, MO, USA) immediately after collection and centrifuged (700 × g, 10 min, 4°C) to separate sperm cells (pellet) from seminal plasma. The supernatant seminal plasma was transferred to new microtubes and subjected to a second centrifugation (10,000 × g, 15 min, 4°C), after which the supernatant was stored in cryogenic tubes. The pellet containing sperm cells was washed with 1 mL of PBS buffer in three centrifugations (700 × g, 10 min, 4°C), with the supernatant discarded after each wash. At the end of the process, the washed sperm cells were stored in liquid nitrogen as well (Souza et al. 2012; Rego et al. 2016). These sperm and seminal plasma samples were kept frozen until the start of proteomic analyses.

#### 2.11.1 Strategy for Analysis of Sperm and Seminal Plasma Proteomes

In this study, semen was collected from 12 rams across 11 sessions, at 3‐day intervals, resulting in a total of 132 ejaculates. For proteomic analysis, samples were used to make individual pools. Pool 1 comprised ejaculates obtained from the odd‐numbered collections (collections on days 1, 3, 5, 7, 9, and 11), whereas pool 2 comprised ejaculates from the even‐numbered collections (days 2, 4, 6, 8, and 10). This strategy was applied individually for each ram, resulting in two sperm pools and two seminal plasma pools per animal. For seminal plasma pooling, 20 µL from each ejaculate was used to compose the corresponding pool. For sperm samples, pooling was performed after protein extraction and quantification. In this case, 100 µg of sperm protein from each ejaculate contributed to the final pool. Thus, the materials for proteomic analysis consisted of 24 sperm and 24 seminal plasma pools, corresponding to two pools of each sample type from each of the 12 animals. This pooling strategy preserved the biological individuality of each ram, while reducing technical variability associated with single‐ejaculate analyses, a critical aspect for robust label‐free quantitative proteomics.

#### 2.11.2 Extraction of Sperm Proteins

Sperm samples previously frozen for proteomic analysis were thawed at room temperature and processed individually for protein extraction. Each sample was resuspended in 300 µL of lysis buffer (8 M urea, 0.075 M NaCl, 0.05 M Tris, 0.2 M sodium orthovanadate, 0.2 M β‐glycerophosphate, protease inhibitors, Sigma, USA; pH 8.2) and incubated on ice with agitation for 15 min. Samples were then sonicated every 2 min for 10 min on ice, with this cycle repeated three times with 2‐min intervals. Subsequently, samples were centrifuged (18,000 × g, 15 min, 4°C), and the supernatant was aliquoted: 3 µL were reserved for protein quantification, and the remaining volume was stored at –80°C until use (Otávio et al. 2023). The 3 µL aliquot was diluted in 27 µL of PBS buffer for determination of total soluble protein concentration using the Bradford method (Bradford 1976), with bovine serum albumin (BSA; Sigma–Aldrich, St. Louis, USA) as standard and readings performed on a microplate spectrophotometer (Epoch Microplate Spectrophotometer; Agilent BioTek, Winooski, VT, USA) (Otávio et al. 2023). After protein quantification, 100 µg of protein from each sample was used to make sperm pools 1 and 2 as described above.

#### 2.11.3 Precipitation of Seminal Plasma Proteins

Seminal plasma previously frozen for proteomic analysis was thawed at room temperature and, for each animal, pools 1 and 2 were prepared as described above. For the protein precipitation, 80 µL of seminal plasma was mixed with 720 µL of cold acetone (−12°C), maintaining a 1:10 (v/v) ratio between sample and solvent (Gomes et al. 2020). The mixture was kept under refrigeration overnight to allow complete protein precipitation. On the following day, samples were centrifuged at 12,000 × g for 30 min (4°C). The supernatants containing acetone were carefully pipetted out, and the resulting protein pellets were subjected to drying (SpeedVac‐Concentrator plus Vacufuge, Eppendorf; D‐HV mode: high‐volatile dry) for 15 min to evaporate any residual acetone. After drying, the pellets were resuspended in 50 µL of ammonium bicarbonate buffer (Ambic, ReagentPlus Sigma‐Aldrich, USA), homogenized for 10 s. and subsequently stored at −20°C until further steps of protein quantification and tryptic digestion.

#### 2.11.4 Sperm and Seminal Plasma Protein Digestion and Peptide Desalting

Sperm and seminal plasma proteins (100 µg) were treated with 10 µL of 8 M urea to initiate denaturation. Briefly, DTT (dithiothreitol) was added to reach a final concentration of 5 mM, followed by incubation at 56°C for 25 min. Alkylation was then carried out by adding IAA (iodoacetamide) to a final concentration of 14 mM, with incubation at 21°C in the dark for 30 min. Prior to digestion, DTT was added again to achieve a final concentration of 5 mM in a final volume of 21 µL. Samples were diluted in 50 mM ammonium bicarbonate at a 1:5 ratio, reducing the urea concentration to 1.6 M. A final concentration of 1 mM calcium chloride was added, and proteins were digested with trypsin (Promega, Madison, WI, USA) at an enzyme‐to‐substrate ratio of 1:50 (w/w), with incubation at 37°C for 16 h. The digestion was stopped by adding TFA (trifluoroacetic acid) to a final concentration of 0.4% (Otávio et al. 2023).

Peptides from sperm and seminal plasma samples were subjected to the same desalting protocol, using SEP columns (Sep‐Park tC18, Waters, USA; Viana et al. 2018). Columns were activated with 3 mL of 100% ACN (acetonitrile), followed by equilibration with 1 mL of 50:50 ACN/Milli‐Q water containing 0.1% formic acid and the addition of 3 mL of TFA. Digested samples were applied to the columns, which were washed with 3 mL of 0.1% TFA to remove residual salts. Columns were then equilibrated with 1 mL of 0.1% formic acid. Peptides were eluted with 2 mL of 50:50 ACN/Milli‐Q water containing 0.1% formic acid, followed by a second elution with 1 mL of 80:20 ACN/Milli‐Q water containing 0.1% formic acid. Eluted peptides were vacuum‐dried in a Concentrator plus (Eppendorf, Hauppauge, NY, USA) and stored at –80°C until analysis.

### 2.12 Label‐Free LC‐MS/MS: Sperm Samples

The concentration of tryptic peptides resulting from digestion of sperm proteins was quantified using the Qubit system (Thermo Fisher, Waltham, MA, USA). Tryptic peptides (1 µL) from each sample (from each pool of each animal) were analyzed in a capillary chromatographic system (nano‐UHPLC Dionex Ultimate 3000) coupled to a hybrid quadrupole‐Orbitrap mass spectrometer (Orbitrap Q Exactive Plus, Thermo Scientific, Germany). The solvents used were: solvent A (0.1% formic acid in water) and solvent B (acetonitrile/0.1% formic acid, 80:20). Technical duplicates were run for each sperm sample.

The chromatographic system consisted of two columns. Initially, peptides were loaded onto a pre‐column Acclaim PepMap 100 C18 (300 µm × 5 mm, 5 µm spherical silica particles, 100 Å pore size; Thermo Fisher, USA) at a flow rate of 5 µL/min with 98% solvent A and 2% solvent B. Peptides were then separated on an analytical column PicoCHIP C18 (75 µm × 25 cm, 15 µm tip; New Objective, USA), packed with Reprosil‐PUR C18 particles (1.9 µm, 120 Å). Elution was carried out using a gradient of solvent B: 2%–40% over 170 min; 40%–85% over 15 min; an isocratic step at 85% for 5 min; followed by re‐equilibration at 2% for 19.5 min. The interface between the nano‐LC system and the mass spectrometer was controlled automatically by Xcalibur 4.1.31.9 software (Thermo Scientific, USA). Peptide ionization was performed with a PicoCHIP nanoelectrospray ion source (New Objective, USA) at a spray voltage of 2.5 kV and a capillary transfer temperature of 250°C. Mass spectra were acquired in positive mode using data‐dependent acquisition (DDA). The DDA cycle consisted of a full scan from 200 to 2000 m/z at a resolution of 140,000 FWHM at m/z 400, with an AGC target of 3 × 10^6^ ions and a maximum injection time of 45 ms. This was followed by MS/MS fragmentation of the 20 most abundant precursor ions using higher‐energy collisional dissociation at 30% collision energy, with a resolution of 17,500 FWHM at m/z 400, an AGC target of 1 × 10^6^ ions, a minimum AGC of 8 × 10^3^ ions, and a maximum injection time of 60 ms. Fragmented precursor ions were dynamically excluded for 10 s (Otávio et al. 2023).

#### 2.12.1 Label‐Free LC‐MS/MS: Seminal Plasma Samples

The analyses of seminal plasma peptides were performed using a high‐resolution mass spectrometry system SYNAPT XS HDMA (Waters, Manchester, UK) coupled to a nano ACQUITY UPLC M‐Class liquid chromatography system (Waters, Milford, USA). For chromatographic separation of the tryptic peptides, a reverse‐phase column HSS T3 C18 (100 Å, 1.8 µm, 75 µm × 150 mm, Waters) was used, maintained at 37°C under a flow rate of 300 nL/min. The elution gradient was established between 0% and 85% acetonitrile (v/v) in 0.1% formic acid for 112 min. The mobile phase A consisted of 0.1% formic acid in water, and mobile phase B consisted of 0.1% formic acid in acetonitrile. Injection was performed with 1 µL of the tryptic digestion, containing approximately 1 µg of peptides. Peptide elution was directly coupled to the electrospray ionization (ESI) source operated in positive mode (ESI+) using the nanoLockSpray system, which allows simultaneous infusion of the calibration standard and the analytical sample. Every 30 s, the analytical channel was interrupted for passage of the reference ion, ensuring mass calibration stability throughout the entire acquisition. Technical duplicates were run for each seminal plasma sample.

### 2.13 Sperm Protein Identification and Statistical Analysis

The analysis of LC‐MS/MS spectra was performed using PatternLab for Proteomics 5.0.0.207 (Carvalho et al. 2016; Passos et al. 2022) with the Comet search engine (version 2019.01). Protein sequences of *Ovis aries* were retrieved from the UniProtKB/Swiss‐Prot database (proteome UP000002356) and complemented with a reversed (decoy) database and common contaminants (bovine serum albumin, keratin, ubiquitin, trypsin). Searches were carried out under high‐resolution settings for both MS and MS/MS stages (High–High, e.g., Q‐Exactive), with a mass tolerance of 35 ppm for precursor ions and 0.02 Da for fragments, organized in bins of 1.0005 m/z. The peptide mass window was defined between 500 and 6000 Da. Digestion was set as fully tryptic, allowing up to two missed cleavages per peptide. Fixed and variable modifications included cysteine carbamidomethylation (fixed) and methionine oxidation (variable), permitting up to two variable modifications per peptide. Validation was performed using the SEPro module, applying a Bayesian discriminator with a cutoff adjusted to accept a 1% FDR. Only spectra with mass error < 6 ppm and peptides containing at least six amino acid residues were retained.

The mass spectrometer generated two.raw files (technical replicate outputs) for each pool, and results from these files were automatically averaged by PatternLab software. Contaminants, reverse and uncharacterized proteins were excluded from this protein list. Then, calculations for pools 1 and 2 were transferred from PatternLab to datasheets and averaged to give the final protein abundances for each sperm sample. Statistical comparison of sperm protein abundances in HF and LF rams was carried out using Student's *t*‐test. Subsequently, the TFold tool in PatternLab was employed to select the differentially abundant proteins (DAPs) using the following cutoff criteria: *q* < 0.05 (Benjamini–Hochberg correction), fold change log_2_ ≥ ±1.5, and *F*‐stringency ≥ 0.22.

#### 2.13.1 Seminal Plasma Protein Identification and Statistical Analysis

The raw LC‐MS/MS data files (.raw) generated from seminal plasma analysis were processed and identified using the Progenesis QI for Proteomics software version 2.0 (Nonlinear Dynamics/Waters, UK) for feature detection, retention time alignment, and relative abundance quantification. Chromatographic alignment and data normalization were automatically performed by the internal algorithm of Progenesis. Organization of mass spectrometer.raw files (technical replicate outputs) for each pool, exclusion of contaminants, reverse and uncharacterized proteins, and definition of the final protein abundances for each seminal plasma sample followed the same strategy described for sperm samples.

Peptide and protein identification was performed by database searching against UniProtKB/Swiss‐Prot, restricted to the reviewed *O. aries* proteome (UP000002356). The search parameters were set for trypsin digestion, allowing up to two missed cleavages, with a maximum protein mass limit of 600 kDa. Fixed and variable modifications included cysteine carbamidomethylation and methionine oxidation, respectively. Quality control of protein identification was based on an FDR < 1%, obtained through a decoy database search. Only proteins identified by at least two unique peptides, each supported by at least two fragment ions and a minimum of five fragment spectra, were accepted. Normalized abundances for each identified protein were automatically calculated by Progenesis, allowing quantitative comparison between HF and LF groups. Label‐free quantification was carried out using the high normalization method, which computes the relative intensities of normalized peptide peaks among samples. For selection of DAPs, normalized abundance values were subjected to ANOVA, considering significance at *p* ≤ 0.05, with a minimum fold change of ≥ 1.3. The final list of DAPs was filtered to remove contaminants and uncharacterized proteins.

### 2.14 Principal Component Analysis (PCA)

The label‐free protein expression matrices from both sperm and seminal plasma datasets, containing normalized intensities exported from PatternLab for Proteomics (sperm) and from Progenesis QI for Proteomics (seminal plasma), were imported into MetaboAnalyst v5—https://www.metaboanalyst.ca/ (Gomes et al. 2020; Passos et al. 2022; Pang et al. 2024). Prior to analysis, dataset integrity was checked, including detection of missing values, zeros, and outliers, removal of technical controls/contaminants, and harmonization of sample and group labels. Zero or undetected values were treated according to the platform's recommended routine: replacement by a small positive value (half of the lowest observed value per variable) to allow logarithmic transformation, followed by k‐NN imputation for variables with few missing entries. Data were then log_2_‐transformed to approximate a normal distribution and stabilize variances, normalized by sample‐wise median scaling to reduce global loading/signal differences, and subsequently autoscaled by variable (mean‐centering and division by standard deviation).

PCA was performed on the standardized matrix (equivalent to correlation‐based PCA), with extraction of scores and loadings for the first two to three components. Explained variance and the scree plot were inspected to evaluate dimensionality. Outlier detection was based on the joint inspection of PC1–PC2 scores and distance/residual diagnostics; samples identified as outliers were excluded only if supported by documented technical error. In addition, 95% confidence ellipses were used solely as a visual aid for cluster inspection.

#### 2.14.1 Analysis of Pathways and Protein Functional Clusters

Functional enrichment analysis of sperm and seminal plasma DAPs in HF and LF phenotypes was conducted using Metascape (https://metascape.org/). This tool enabled the semantic aggregation of enriched terms, identification of highly connected subnetworks through the MCODE algorithm, and automatic integration with multiple public databases, including Gene Ontology (GO), KEGG, Reactome, and DisGeNET. For this purpose, UniProt identifiers were converted into gene names using the ID Mapping tool (https://www.uniprot.org/id-mapping). Enriched terms, pathways, and functional clusters were identified according to statistically significant *p* value and false discovery rate (FDR) thresholds defined by the database (Bezerra et al. 2019; Milacic et al. 2024).

## 3 Results

### 3.1 Characterization of Rams With HF and LF

Considering all 12 animals and 11 ejaculates obtained in the experiment, the average TMR was 90.58% ± 0.75% in fresh semen and 56.46% ± 2.87% in frozen‐thawed semen, with TMR reduction calculated as 33.48% ± 2.27%. Thus, HF rams (*n* = 5) had a TMR of 26.22% ± 2.39% (TMR reduced from 91.87% ± 0.63% in fresh to 65.65% ± 2.58% in frozen‐thawed semen). As a contrast, LF rams (*n* = 7) averaged much higher (*p* = 0.006) TMR value (37.70% ± 2.23%), as TMR decreased from 89.47% ± 1.15% in fresh to 50.05% ± 2.33% in frozen‐thawed semen. Sperm PM decreased less in HF rams (from 87.20% ± 0.50% to 58.12% ± 2.82%) than in LF rams (from 85.54% ± 1.41% to 44.00% ± 2.13%) after semen cryopreservation (*p* = 0.006). Rams with the HF phenotype had better plasma membrane (39.00% ± 2.79% vs. 15.14% ± 0.92%; *p* = 0.0009) and acrosome integrity (37.54% ± 2.72% vs. 15.40% ± 1.92%; *p* = 0.0001) compared with LF rams. Fresh semen of rams categorized as HF and LF phenotypes had similar values of TMR (*p* = 0.100) and sperm PM (*p* = 0.369; Table 1).

**Table 1:** Kinetic and functional parameters of fresh and frozen‐thawed sperm from rams with high (HF) and low (LF) semen freezability. Values represent the average (±SEM) of 11 ejaculates collected from 12 rams at 3‐day intervals.

Parameter	Fresh semen					Frozen‐thawed semen					Fresh/frozen‐thawed Δ in HF rams [1–3]		Fresh/frozen‐thawed Δ in LF rams [2–4]		
	HF [1]		LF [2]		*p* [1 vs. 2]	HF [3]		LF [4]		*p* [3 vs. 4]					*p* [1–3] vs. [2–4]
	Mean	SEM	Mean	SEM		Mean	SEM	Mean	SEM		Mean	SEM	Mean	SEM	
TM (%)	91.87	0.63	89.47	1.15	0.100	65.65	2.58	50.05	2.33	0.002	26.22^a^	2.39	37.70^a^	2.23	0.006
PM (%)	87.20	0.50	85.54	1.41	0.369	58.12	2.82	44.00	2.13	0.002	29.09	2.74	41.54	1.34	0.006
MI (%)	—	—	—	—	—	39.00	2.79	15.14	0.92	< 0.001	—	—	—	—	—
AI (%)	—	—	—	—	—	37.54	2.72	15.40	1.92	< 0.001	—	—	—	—	—

Abbreviations: Δ, variation; AI, acrosome integrity; HF, high semen freezability; LF, low semen freezability; MI, membrane integrity; PM, sperm progressive motility; TM, total sperm motility; TMR, total sperm motility reduction.

^a^ Animals with TMR below and above the overall average TMR (33.5% ± 2.3%) were grouped in HF and LF phenotypes, respectively.

Sperm viability in frozen‐thawed semen from HF (46.00% ± 2.53%) and LF rams (38.86% ± 2.53%) did not different (*p* = 0.2132; Figure 1), neither did normal morphology, major defects, or DNA integrity between the two groups. Mitochondrial membrane potential evaluated in frozen‐thawed sperm of HF (50.60% ± 3.90%) and LF rams (41.71% ± 1.93%) were equivalent (*p* = 0.1481; Figure 2). All parameters without differences (*p* > 0.05) between HF and LF rams are summarized in Table 2.

**Figure 1:**
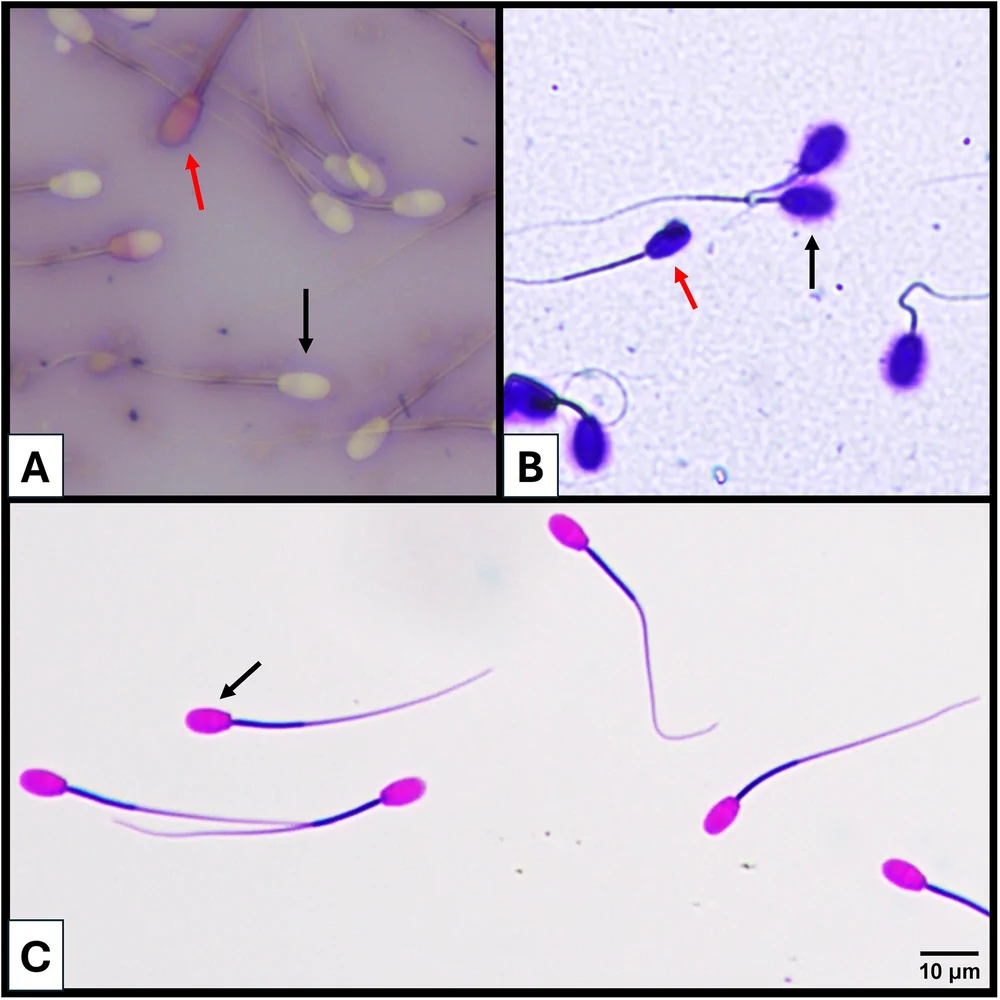
Morphofunctional parameters of frozen‐thawed ram spermatozoa. (A) Sperm viability based on eosin‐nigrosin staining. Black and red arrows indicate viable (unstained) and dead sperm (stained), respectively. (B) Sperm chromatin integrity analyzed by the sperm chromatin dispersion test. Black and red arrows point to a cell with intact chromatin (presence of a halo) and a cell with fragmented chromatin (absence of a halo), respectively. (C) Sperm morphology evaluation using rose bengal staining, with the black arrow indicating a sperm cell with normal morphology. The scale bar in the last panel represents 10 µm.

**Figure 2:**
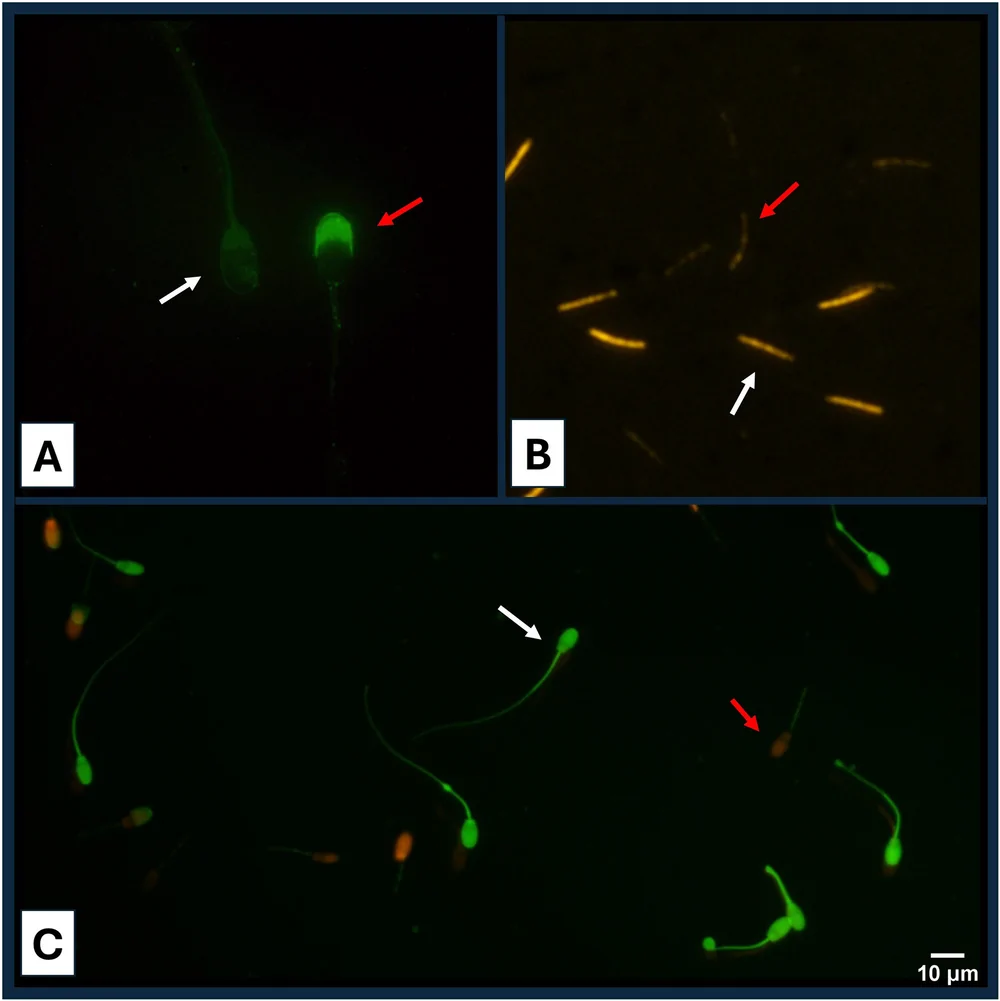
Functional assessment of frozen‐thawed ram spermatozoa using fluorescent probes. (A) Sperm acrosome labeling with the FITC‐PNA probe. White and red arrows indicate sperm with intact (absence of fluorescence in the acrosomal region) and reacted acrosome (green fluorescence), respectively. (B) Analysis of mitochondrial membrane potential using the JC‐1 probe. White and red arrows point to spermatozoa with high (bright orange fluorescence) and low mitochondrial potential (less intense or absent fluorescence), respectively. (C) Evaluation of plasma membrane integrity using IP/DCFDA. White and red arrows indicate sperm with intact membranes (green fluorescence) and damaged membranes (red fluorescence), respectively. The scale bar in the last panel represents 10 µm.

**Table 2:** Sperm parameters of fresh and frozen‐thawed semen that did not show significant differences between rams with high (HF) and low (LF) semen freezability (*p* > 0.05). Values represent the average (±SEM) of 11 ejaculates collected from 12 rams at 3‐day intervals.

Parameter	Fresh semen				Frozen‐thawed semen			
	HF		LF		HF		LF	
	Mean	SEM	Mean	SEM	Mean	SEM	Mean	SEM
VSL (μm/s)	94.80	2.98	93.40	1.54	48.72	1.82	47.94	1.29
VCL (μm/s)	212.82	3.87	209.33	3.49	144.92	3.71	139.24	2.79
VAP (μm/s)	116.10	3.16	109.16	2.36	63.58	2.13	59.53	0.98
BCF (Hz)	17.06	0.13	16.91	0.35	12.76	0.18	12.10	0.18
ALH (μm)	4.14	0.05	4.34	0.15	3.36	0.06	3.24	0.05
LIN (VSL/VCL)	0.44	0.01	0.46	0.01	0.36	0.01	0.37	0.01
STR (VSL/VAP)	0.80	0.00	0.80	0.00	0.70	0.00	0.70	0.00
HAC (rad)	0.56	0.01	0.56	0.01	0.40	0.01	0.41	0.01
DCL (μm)	59.98	0.59	59.90	0.92	42.12	0.68	41.59	0.68
DSL (μm)	25.32	0.61	25.87	0.56	14.18	0.36	13.83	0.36
Average path distance (μm)	30.94	0.59	30.74	0.64	19.04	0.37	18.68	0.37
Viability (%)	79.00	1.40	78.43	1.29	46.00	2.53	38.86	2.53
Normal M (%)	98.62	0.26	98.43	0.23	96.33	0.87	94,86	0.57
Major D (%)	0.20	0.13	0.57	0.23	0.33	0.21	0.57	0.20
Minor D (%)	1.20	0,01	1.00	0.01	3.27	0.82	4.57	0.49
CI (%)	93.60	0.97	96.86	0.70	70.60	3.01	68.86	3.33
MMP (%)	—	—	—	—	50.60	3.90	41.71	1.93

Abbreviations: ALH, amplitude of lateral head displacement; BCF, beat‐cross frequency; CI, chromatin integrity; DCL, distance curvilinear line; DSL, distance straight line; HAC, head angular change; LIN, linearity (VSL/VCL); Major D, major defects; Minor D, minor defects; MMP, mitochondrial membrane potential; Normal M, normal morphology; STR, straightness (VSL/VAP); VAP, average path velocity; VCL, curvilinear velocity; Viability, sperm viability; VSL, straight‐line velocity.

#### 3.1.1 Global Proteomic Profile of Sperm and Seminal Plasma From HF and LF Rams

Mass spectrometry and data analysis allowed the identification of 1853 proteins in the sperm samples (Supporting Information S1), being 1599 and 1677 proteins in the sperm of HF and LF rams, respectively, with 1423 shared proteins by the two groups of animals (Figure 3A). There were 1127 proteins identified in the ram seminal plasma (Supporting Information S2), where all proteins were shared by HF and LF rams, with no exclusive proteins observed in either group (Figure 3B).

**Figure 3:**
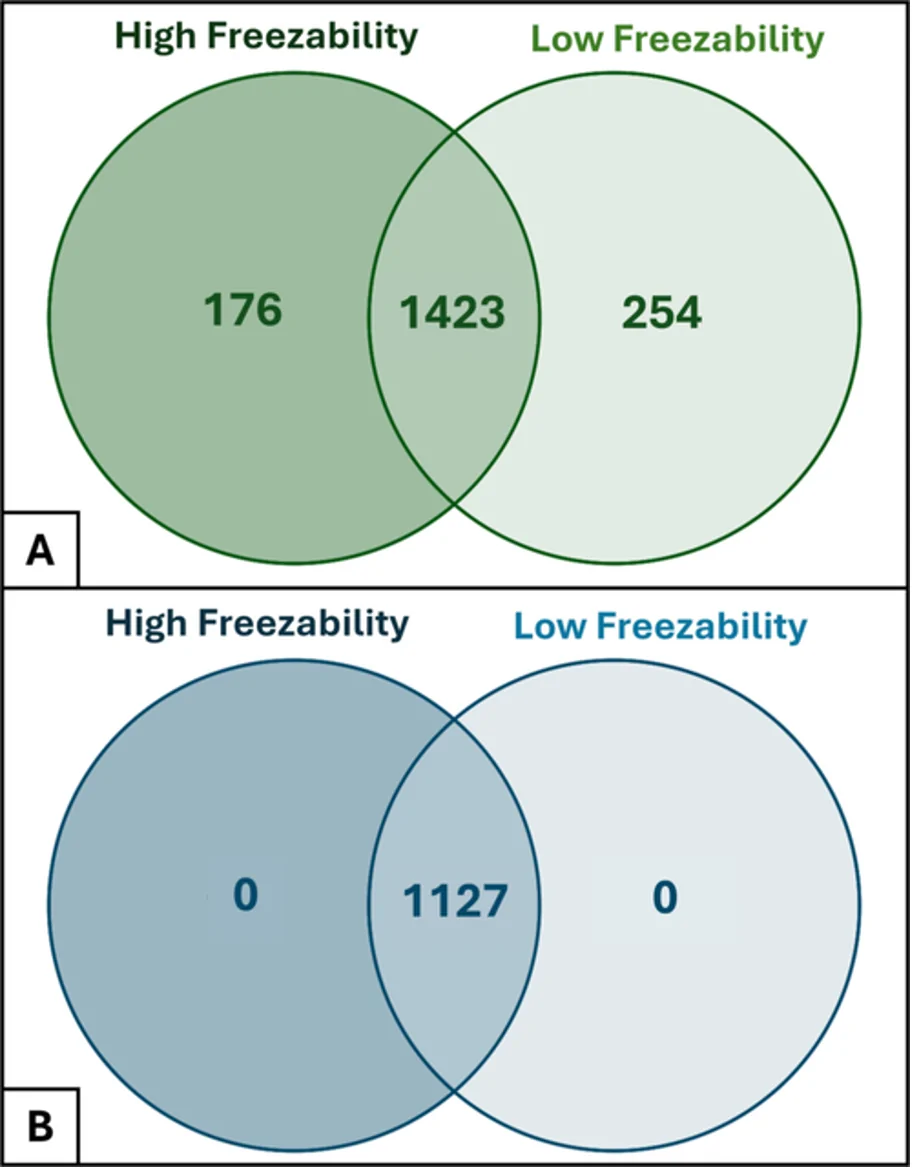
Venn diagrams representing the number of proteins identified in rams with high (HF) and low (LF) semen freezability. (A) Sperm proteins. (B) Seminal plasma proteins. Prepared with Venn software v2.1.

PCA of sperm protein abundances revealed partial overlap between HF and LF groups, with principal component 1 (PC1) explaining 14.7% of the variance and principal component 2 (PC2) explaining 8.6% (Figure 4A). Similarly, PCA of seminal plasma protein abundances indicated a clear trend toward group separation, with PC1 explaining 26.4% of the variance and PC2 explaining 20.1%, showing partial but consistent discrimination between HF and LF groups (Figure 4B).

**Figure 4:**
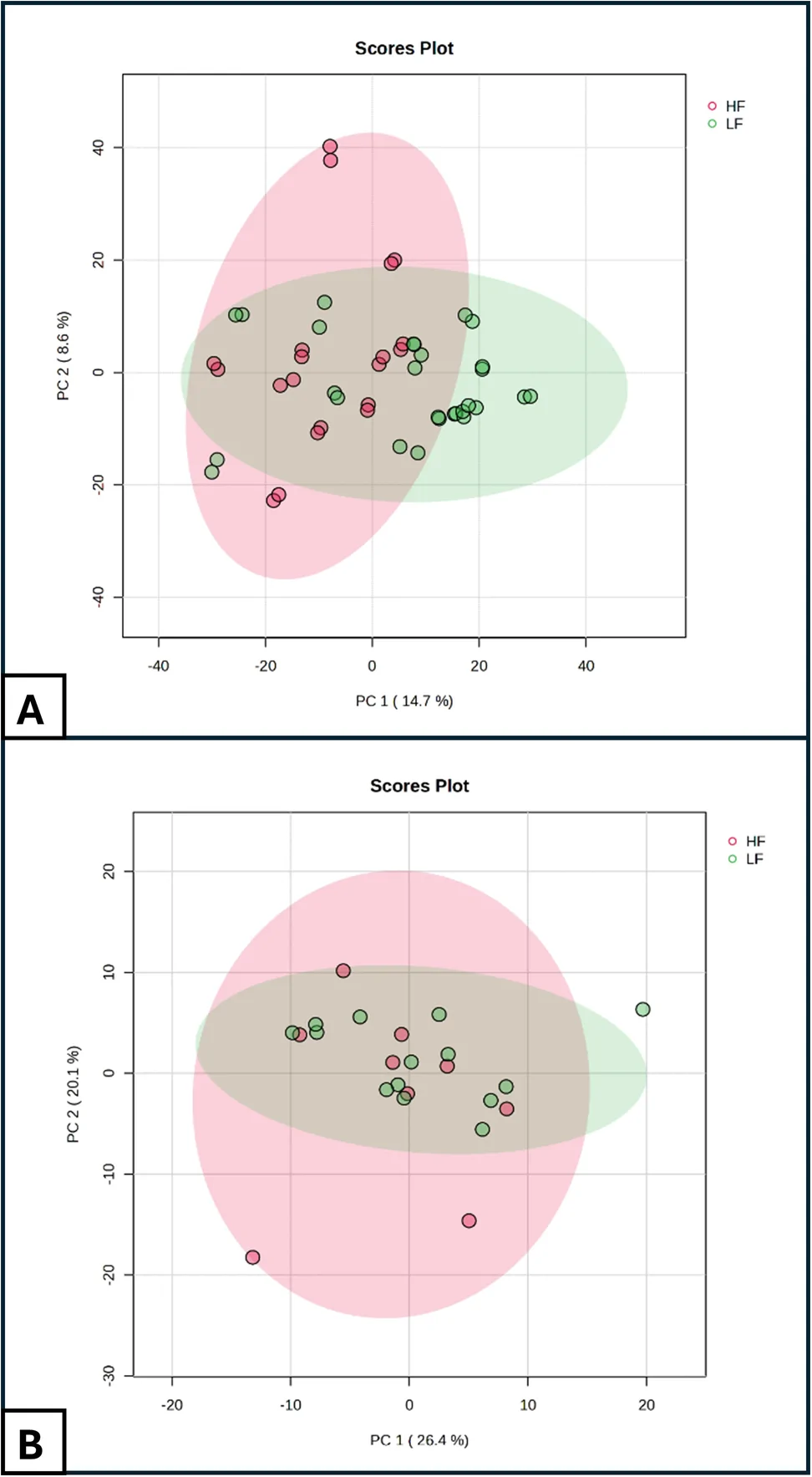
Principal component analysis (PCA) considering sperm and seminal plasma differentially abundant proteins identified in rams with high (HF) and low (LF) semen freezability. (A) PCA of sperm proteins, where PC1 and PC2 explain 14.7% and 8.6% of the variance, respectively, with partial overlap between HF and LF groups. (B) PCA of seminal plasma proteins, where PC1 and PC2 explain 26.4% and 20.1% of the variance, respectively, showing partial separation between HF and LF groups.

#### 3.1.2 Identification and Functional Analyses of DAPs in Spermatozoa From HF and LF Rams

There were 149 DAPs in the ram sperm cells, with 73 and 76 proteins more abundant in HF and LF sperm, respectively. Sperm proteins such as 5 kDa bactenecin, cathelicidin‐2, N(4)‐(beta‐N‐acetylglucosaminyl)‐L‐asparaginase, transcobalamin‐2, and cathelicidin‐1 had the highest fold change in the HF group. Proteins such as cilia‐ and flagella‐associated protein 69, DCC‐interacting protein 13‐alpha, dynein regulatory complex protein 9, cytochrome b5 domain‐containing protein 1, and cytochrome b‐c1 complex subunit 1, mitochondrial, appeared with the highest fold change in LF rams (Table 3).

**Table 3:** List of the 30 most differentially abundant proteins (DAPs) and respective fold changes in the sperm of rams with high (HF) and low (LF) semen freezability. Proteins were selected based on PatternLab´s TFold module (*q* < 0.05; log_2_FC ≥ ± 1.2; F‐stringency ≥ 0.22).

Uniprot accession	Phenotype	Fold change	*p*	Protein
W5NV75	HF	5.79	0.018	5 kDa bactenecin
P79362	HF	5.79	0.018	Cathelicidin‐2
W5PA90	HF	5.28	0.006	N(4)‐(Beta‐N‐acetylglucosaminyl)‐L‐asparaginase
W5PEY4	HF	4.14	< 0.001	Transcobalamin‐2
P54230	HF	4.13	0.040	Cathelicidin‐1
W5QGZ7	HF	3.53	0.008	Trans‐Golgi network protein 2
W5PJY6	HF	2.95	0.043	ADAM metallopeptidase domain 28
W5PS94	HF	2.87	0.002	Nucleobindin‐1
W5P627	HF	2.85	0.013	Gelsolin
W5PSZ5	HF	2.80	0.045	Annexin
A0A6P3TBL3	HF	2.80	0.002	Hypoxia up‐regulated protein 1
W5PPU0	HF	2.76	0.039	Sepiapterin reductase
W5P0V6	HF	2.70	0.008	Saccharopine dehydrogenase‐like oxidoreductase
W5PVZ9	HF	2.62	0.021	Dystroglycan 1
W5NXV7	HF	2.49	0.015	Patched domain containing 3
W5NTL9	LF	2.44	0.012	Cytochrome c oxidase subunit 6B1
W5PX31	LF	2.49	0.026	Spermatogenesis‐associated protein 19, mitochondrial
A0A6P7DVD9	LF	2.53	0.013	Trifunctional enzyme subunit beta, mitochondrial
W5PKE4	LF	2.59	0.009	Aldo‐keto reductase family 1 member B1
W5PDC1	LF	2.61	0.009	glycerol kinase
A0A8V6WE19	LF	2.67	0.022	NADH dehydrogenase [ubiquinone] 1 alpha subcomplex subunit 2
W5Q295	LF	2.69	0.035	Cilia‐ and flagella‐associated protein 161
W5P6Y6	LF	2.70	0.006	Adenylate kinase 8
W5P5P7	LF	2.75	0.008	Myb‐like domain‐containing protein
W5PHL2	LF	2.85	0.006	Lipocalin/cytosolic fatty‐acid binding domain‐containing protein
W5Q5G6	LF	2.95	0.022	Cytochrome b‐c1 complex subunit 1, mitochondrial
W5Q8L2	LF	3.31	0.032	Cytochrome b5 domain‐containing protein 1
W5QGB0	LF	3.88	0.003	Dynein regulatory complex protein 9
W5PYQ5	LF	3.90	0.010	DCC‐interacting protein 13‐alpha
W5PR77	LF	4.01	0.009	Cilia‐ and flagella‐associated protein 69 ARM repeats domain‐containing protein

Functional analyses of HF ram sperm proteins identified the enrichment of protein folding, post‐translational protein phosphorylation, positive regulation of cell cycle process, actin filament organization, among other pathways (Figure 5A). Sperm proteins that were predominant in LF rams were mainly linked to cilium movement, glycolysis, pyruvate metabolism, spermatid development, fatty acid beta oxidation, and fertilization, among other pathways and mechanisms (Figure 5B). According to the Metascape platform, proteins with greater abundances in the sperm of LF rams were interconnected in three mains complexes, defined as glycolysis/gluconeogenesis, pyruvate metabolism and aerobic respiration; axonemal dynein complex assembly, motor proteins. and cilium movement; sperm flagellar motility, sperm motility and cilium movement in motile cells (Figure 6).

**Figure 5:**
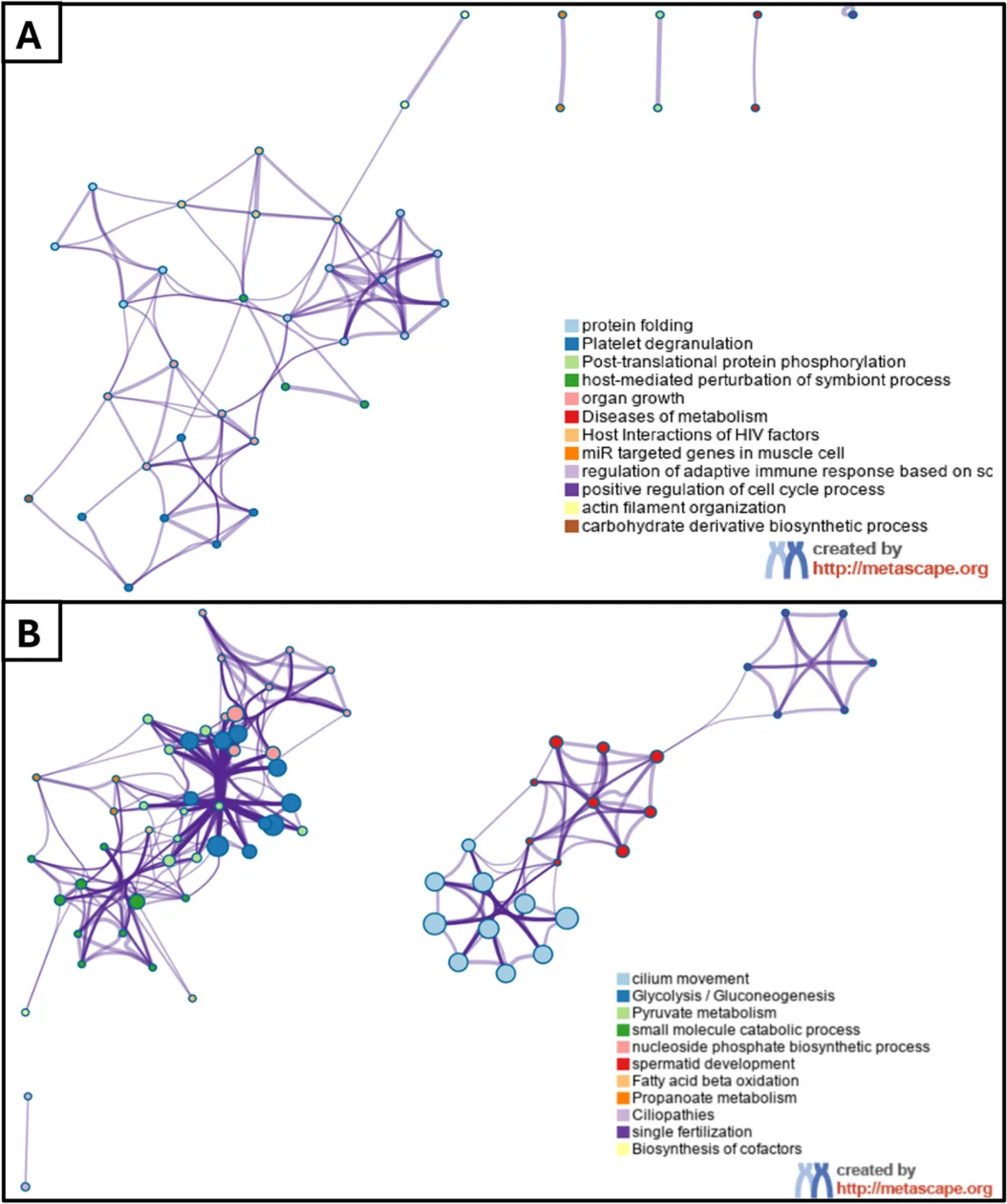
Network of enriched terms and pathways associated with the proteins with greater abundances in the sperm of rams with high (A: HF; 73 proteins) and low (B: LF; 76 proteins) semen freezability. Analyses were conducted using Metascape (https://metascape.org/gp/index.html). Each node represents an enrichment term (gene ontology or pathway) and is color‐coded according to its functional category. Edges indicate similarity between terms based on gene overlap.

**Figure 6:**
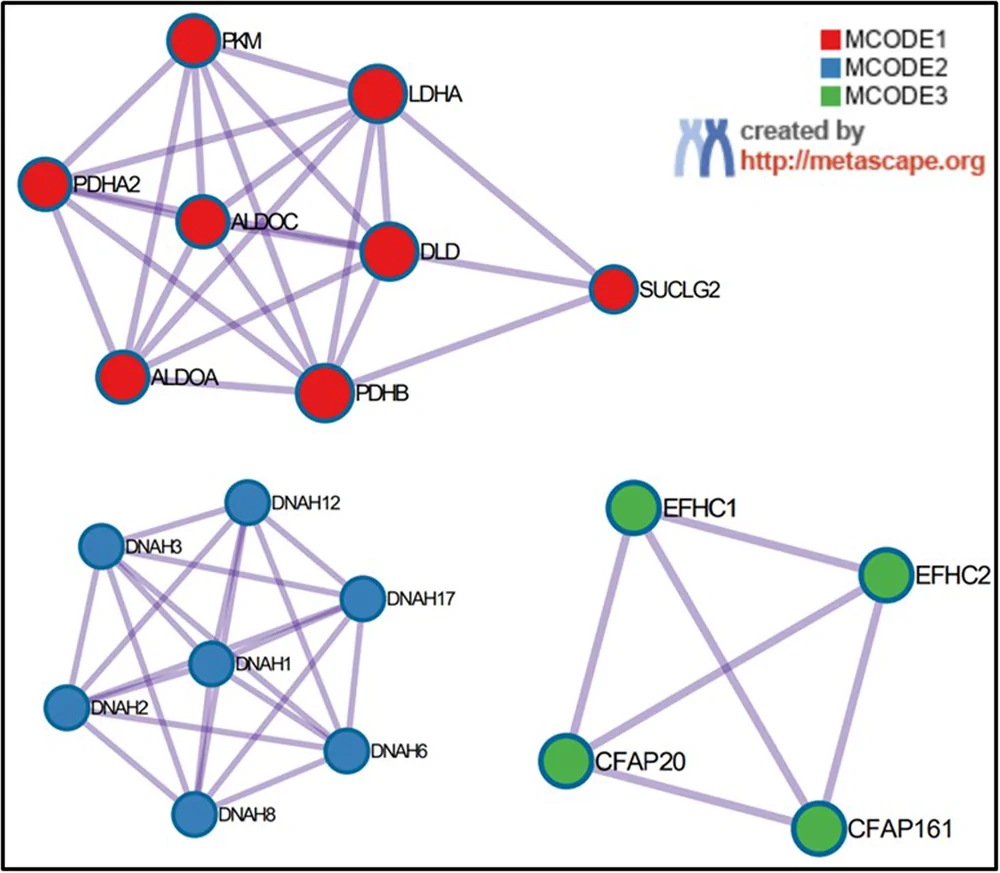
Protein complexes (MCODEs) identified in the interaction network of the 76 differentially abundant proteins in the sperm of rams with low semen freezability (LF). Analysis was conducted with Metascape (https://metascape.org/gp/index.html). MCODE_1 (red) is associated with glycolysis/gluconeogenesis processes, pyruvate metabolism, and aerobic respiration. MCODE_2 (blue) is related to axonemal dynein complex assembly, motor proteins, and cilium movement. MCODE_3 (green) involves sperm flagellar motility. Sperm motility and cilium movement in motile cells.

#### 3.1.3 Identification and Functional Analyses of DAPs in Seminal Plasma From HF and LF Rams

There were 25 and 45 proteins with greater abundances in the seminal plasma of HF and LF rams, respectively. Proteins showing higher abundance in HF seminal plasma were linked to antioxidant protection, protein folding, and epididymal interactions, including beta‐defensin, S100 proteins, epididymal sperm‐binding protein 1 (ESBP1), T‐complex protein 1 subunit zeta, 14‐3‐3 domain‐containing protein, proteasome subunit alpha type, and aminopeptidase. Proteins with greater abundance in the seminal plasma of LF rams included heat shock 70 kDa protein 13, haptoglobin, and mitochondrial subunits such as cytochrome c oxidase subunit 4 and NADH dehydrogenase subunit 12. These proteins were mainly associated with cellular stress responses, antioxidant activity, and mitochondrial metabolism (Table 4).

**Table 4:** List of all differentially abundant proteins (DAPs) and respective fold changes in the seminal plasma of rams with high (HF) and low (LF) semen freezability. Proteins were selected based on the criteria established in Progenesis QI for Proteomics (*p* < 0.05; FC ≥ ± 1.3).

Uniprot accession	Phenotype	Fold change	*p*	Protein
A0A835ZJG6	HF	2.40	0.035	Beta‐defensin
W5PU33	HF	2.25	0.010	T‐complex protein 1 subunit zeta
A0A6P3EH65	HF	1.97	0.006	Trafficking protein particle complex subunit
A0A6P3EGD4	HF	1.65	0.048	Angiopoietin‐1
A0A836ABN1	HF	1.58	0.015	Inorganic diphosphatase
W5QIV1	HF	1.54	0.022	Protein S100
A0A835ZW78	HF	1.54	0.041	WAP four‐disulfide core domain protein 13
W5PU09	HF	1.50	0.009	Protein S100 (S100G)
A0A836A6L8	HF	1.50	0.000	Small monomeric GTPase
A0A6P7EKL5	HF	1.49	0.048	Ephrin‐A1
A0A836A9T1	HF	1.48	0.048	Glutaminyl‐peptide cyclotransferase
W5PZF1	HF	1.43	0.035	SCP domain‐containing protein (GLIPR1L1)
A0A836CZC7	HF	1.43	0.002	Protein FAM184A/B N‐terminal domain‐containing
A0A835ZPE6	HF	1.40	0.024	Mis18‐binding protein 1
A0A835ZRQ6	HF	1.37	0.011	FeS cluster biogenesis domain‐containing protein
W5QAZ0	HF	1.37	0.007	Phosphoribosyl pyrophosphate synthase‐associated protein 2
A0A6P7EVV0	HF	1.37	0.028	Epididymal sperm‐binding protein 1
Q6QAT4	HF	1.36	0.000	Beta‐2‐microglobulin
A0A836A0S9	HF	1.34	0.026	14‐3‐3 domain‐containing protein
W5QIY3	HF	1.33	0.002	Proteasome subunit alpha type (PSMA3)
A0A836D5L1	HF	1.29	0.040	Phosphopyruvate hydratase (Enolase)
A0A836A142	HF	1.27	0.009	Aminopeptidase
A0A6P7EN74	HF	1.26	0.002	Guanine nucleotide‐binding protein G(s) subunit alpha
W5PFV5	HF	1.26	0.009	Aminopeptidase (NPEPPS)
A0A6P3E767	HF	1.25	0.008	Protein S100
A0A836D2V4	LF	1.25	0.015	Prostaglandin‐H2 D‐isomerase
W5NVM0	LF	1.25	0.015	Disintegrin and metalloproteinase domain‐containing protein 2
A0A835ZKZ2	LF	1.25	0.047	Cystatin domain‐containing protein
W5Q411	LF	1.25	0.001	Heat shock 70 kDa protein 13
W5NTB3	LF	1.26	0.002	Cystatin domain‐containing protein (CST6)
W5P6G0	LF	1.26	0.003	26S proteasome non‐ATPase regulatory subunit 8
A0A836AGQ1	LF	1.26	0.016	ABC transporter domain‐containing protein
A0A836ADE6	LF	1.27	0.003	Unconventional myosin‐VIIb
A0A836AD58	LF	1.27	0.046	J domain‐containing protein
W5PVP5	LF	1.28	0.026	Adenine phosphoribosyltransferase
W5P0G8	LF	1.28	0.005	Prefoldin subunit 3
W5PT38	LF	1.28	0.000	Histone H2A
A0A836CUQ3	LF	1.29	0.023	Mirror‐image polydactyly 1 (fragment)
A0A6P3TCX5	LF	1.29	0.012	m7GpppX diphosphatase
Q9XSM0	LF	1.29	0.013	Prostaglandin‐H2 D‐isomerase
A0A835ZUL5	LF	1.30	0.007	Secretoglobin family 1D member
W5P8K0	LF	1.30	0.027	Dynactin subunit 2
W5Q0G8	LF	1.30	0.016	Inositol‐1‐monophosphatase
W5P455	LF	1.32	0.001	BOS complex subunit TMEM147
A0A6P7E1T2	LF	1.33	0.000	Carboxypeptidase A1
A0A835ZRB5	LF	1.33	0.010	C‐type lectin domain‐containing protein
W5PUH4	LF	1.33	0.007	Glutathione transferase
W5PGV9	LF	1.33	0.002	Maestro heat‐like repeat family member 2B
A0A6P3ED33	LF	1.33	0.004	Dynactin subunit 4
A0A836CTD5	LF	1.34	0.001	UDP‐N‐acetylglucosamine
W5NPM9	LF	1.34	0.017	Purine‐rich element‐binding protein gamma
A0A836D5W4	LF	1.36	0.005	Copper transport protein ATOX1
A0A836AEH3	LF	1.36	0.004	HEAT repeat‐containing protein 4
A0A6P3E8V7	LF	1.37	0.035	Alsin
A0A836CYI1	LF	1.42	0.003	Carboxypeptidase
A0A835ZT99	LF	1.43	0.017	Plasminogen activator inhibitor 1
W5PJT9	LF	1.43	0.032	IF rod domain‐containing protein
A0A835ZT30	LF	1.47	0.003	Globin domain‐containing protein
A0A6P7DJF4	LF	1.47	0.013	Peripheral plasma membrane protein CASK
A0A6P3EDF3	LF	1.47	0.011	Essential protein Yae1 N‐terminal domain‐containing protein
W5PQI7	LF	1.51	0.000	Prostate and testis expressed protein 2‐like
A0A836A2E1	LF	1.53	0.005	Protein FAM208B
A0A8N7XJ18	LF	1.59	0.000	NADH dehydrogenase [ubiquinone] 1 alpha subcomplex subunit 12
W5P7T9	LF	1.60	0.001	RAB11‐binding protein RELCH
W5P8F9	LF	1.63	0.015	Lipid‐binding serum glycoprotein N‐terminal domain‐containing protein
A0A836D6B3	LF	1.65	0.000	PC4 and SFRS1‐interacting protein
A0A835ZVQ1	LF	1.68	0.000	Importin subunit alpha‐1 (Fragment)
W5PVL5	LF	2.25	0.008	Heat shock 70 kDa protein 4
A0A835ZW29	LF	2.40	0.035	Haptoglobin
A0A6P7EQR6	LF	2.25	0.010	Cytochrome c oxidase subunit 4

Enriched terms and pathways associated with proteins with greater abundance in the seminal plasma of rams with HF were mainly defined as MHC class I‐mediated antigen processing and M phase (Figure 7A), protein folding, cellular responses to stress e small molecule biosynthetic process characterized were the major pathways of LF rams (Figure 7B).

**Figure 7:**
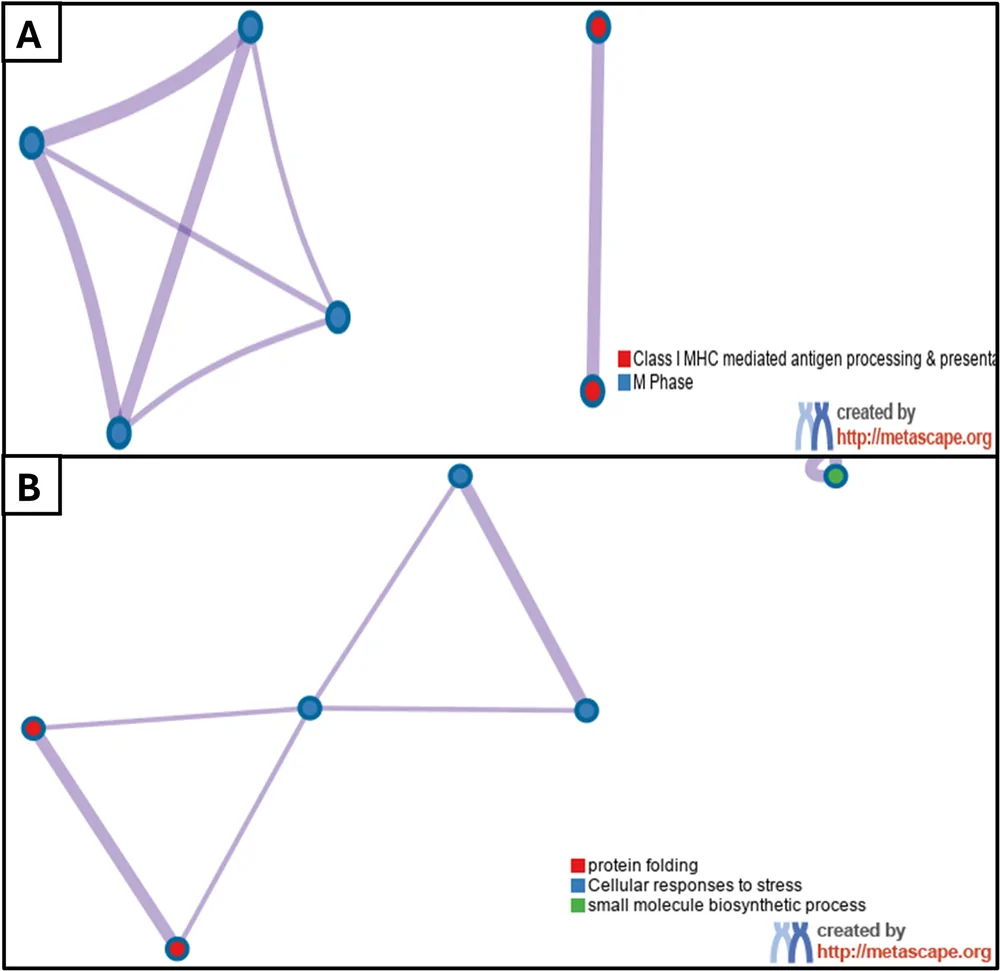
Network of enriched terms and pathways associated with proteins with greater abundance in the seminal plasma of rams with high (A: HF; 25 proteins) and low (B: LF; 23 proteins) semen freezability. Analyses were conducted with Metascape (https://metascape.org/gp/index.html). Each node represents an enrichment term (gene ontology or pathway) and is color‐coded according to its functional category. Edges indicate similarity between terms based on gene overlap.

## 4 Discussion

The present study provides evidence that variations in semen freezability among tropically adapted rams are closely related to the protein composition of sperm and seminal plasma. These findings support the concept that cryoresistance is a multifactorial trait resulting from the balance among structural stability, metabolic competence, and antioxidant or chaperone protection mechanisms in the sperm cells and seminal plasma microenvironment. Parameters of fresh and frozen‐thawed semen presented in our study, as well as proteomic data, were based on ejaculates from 12 rams collected 11 times, within 5 weeks, making a total of 132 ejaculates. This large set of samples provided consistence and reliability of data for the definition of the freezability phenotypes and proteomic analyses. In this context, detailed analyses of sperm and seminal plasma proteome landscapes contribute to better understanding of the molecular determinants of sperm cryoresilience in rams.

### 4.1 Functional Evaluation of Semen and Post‐Thaw Cryopreservation Impact

Semen from HF rams maintained greater TM and progressive sperm motility, as well as better preservation of sperm plasma and acrosomal membrane integrity after cryopreservation.

Post‐thaw sperm TM was used as the primary phenotypic marker of semen freezability because it is one of the most consistently validated indicators of cryotolerance in ruminants. Studies in sheep have demonstrated that rams classified as high‐freezability exhibit significantly higher post‐thaw motility, supporting its use as a reliable indicator of cryoresilience (Zhang et al. 2024b; Vozaf et al. 2022). Similar patterns have been reported in cattle, where post‐thaw motility is routinely adopted to distinguish high‐ and low‐freezability bulls and is associated with mitochondrial function, membrane stability, and overall sperm survival after thawing (Gacem et al. 2023). Reviews on sperm cryobiology further emphasize that post‐thaw motility remains one of the most informative and widely used parameters to evaluate cryopreservation success in mammalian species (Peris‐Frau et al. 2020). Although other traits (such as membrane integrity, acrosome status, mitochondrial potential, and viability) also contribute to the assessment of cryotolerance, motility integrates multiple structural and metabolic processes affected by freezing, making it a biologically meaningful criterion for classifying rams into HF and LF phenotypes.

From a mechanistic perspective, the preservation of cellular and acrosomal membranes is essential for protection against the osmotic and oxidative effects generated during freezing and thawing. These structures are crucial for maintaining ionic homeostasis, intracellular signaling, and fusion with the oocyte, which support sperm fertilizing competence (Harrison and Vickers 1990; Yániz et al. 2013; W. Sun et al. 2020). Studies show that pronounced reductions in sperm motility, viability, and membrane integrity after freezing and thawing are directly associated with decreased fertility in sheep, highlighting the central role of sperm motility and membrane integrity (Yánez‐Ortiz et al. 2022; Bai et al. 2023; Zhang et al. 2024b).

Parameters of fresh semen were similar in HF and LF animals. This means that several methods currently used for evaluation of fresh semen do not have the precise accuracy to meaningfully predict sperm cryotolerance, at least in rams. Individual variations in sperm quality after the challenge of cryopreservation have been reported in bulls (Rego et al. 2016; Gomes et al. 2020; Viana et al. 2025) and rams (Pequeño et al. 2023; Bülbül et al. 2025; Dayanıklı et al. 2025). These variations would be determined by intrinsic molecular and structural factors of sperm and/or seminal plasma that come into play only after a drastic challenge, such as cryopreservation, is applied to the gametes. We present empirical evidence in our study that both sperm and seminal plasma proteomes determined in fresh semen comprise the framework of semen freezability‐determining factors.

#### 4.1.1 Global Proteomic Profile of Sperm and Seminal Plasma From HF and LF Rams

Label‐free mass spectrometry allowed the identification of 1853 sperm proteins in HF and LF rams, respectively, of which 1423 were shared between the two groups. These findings support the existence of a conserved proteomic core common to all animals, along with quantitative differences associated with semen freezability phenotypes. In the seminal plasma, 1127 proteins were identified, all shared by HF and LF rams. This extensive overlap suggests that phenotypic differences are more likely attributable to variation in protein abundances than to the exclusive presence or absence of specific proteins.

The number of proteins identified in both sperm and seminal plasma samples of the present study is consistent with, and in some cases exceeds, values reported in previous publications. For example, Pini et al. (2016) reported 685 proteins in ejaculated ram spermatozoa, Hitit et al. (2021) identified 997 proteins in ram sperm using label‐free LC–MS/MS, and Chen et al. (2024) reported 2050 proteins in a proteomic dataset of ram sperm. For seminal plasma, Druart et al. (2013) described 302 proteins using GeLC‐MS/MS and 2D‐LC‐MS/MS, Soleilhavoup et al. (2014) identified 727 proteins using GeLC‐MS/MS, and Townsend et al. (2025) characterized 829 proteins using LC‐MS/MS. These studies illustrate the expansion of proteomic coverage in ram semen as mass spectrometry platforms, analytical workflows, and bioinformatic tools have advanced. The deeper coverage of the seminal plasma proteome achieved in the present study provides an unprecedented molecular characterization of this fluid and offers new insights into its roles in sperm physiology.

#### 4.1.2 Analysis of DAPs in Spermatozoa From HF and LF Rams

There were 73 and 76 sperm proteins with greater abundances in HF and LF rams, respectively. Cathelicidin 1, Cathelicidin 2, and the 5 kDa bactenecin peptide were among the proteins enriched in the HF group. Cathelicidins are well‐characterized antimicrobial peptides, participating in innate immune defense and protection of reproductive tissues (Avila 2017; Bolosov et al. 2021). Studies have also identified cathelicidins in the seminal plasma of Bos indicus bulls (Rego et al. 2016) and, although their specific roles in ovine spermatozoa remain to be clarified, greater abundance of cathelicidins in HF rams may indicate these individuals have better protective elements to support sperm cryoresilience.

The sperm proteome of high freezability rams was also characterized by greater abundance of Hypoxia up‐regulated protein 1 (HYOU1) and HSP90B1, as well as proteins related to membrane organization and cytoskeletal dynamics, particularly gelsolin, annexin, dystroglycan 1 and ADAM28. In agreement with our study, previous reports indicated that rams with superior sperm cryoresilience had more HYOU1 and HSP90B1, molecules that are associated with antioxidant defense, protection against apoptosis, and maintenance of cellular homeostasis (Ren et al. 2023). Gelsolin redistribution during freezing, thawing, and capacitation in ram spermatozoa indicates a role in actin remodeling (de las Heras et al. 1997), while annexins are associated with membrane stability and sperm quality (Vašíček et al. 2022). Dystroglycan 1 participates in interactions between the plasma membrane and extracellular matrix (Anderson et al. 2024), and ADAM28 is part of a protein family involved in sperm surface remodeling during epididymal maturation (Hunnicutt et al. 1997). Together, sperm of HF rams appeared suited to better cryoresistance because these cells have a portfolio of proteins promoting membrane and cytoskeletal stability, antioxidant defense, and protection against apoptosis.

Proteins with greater abundance in spermatozoa from LF rams were predominantly associated with mitochondrial function, flagellar organization, and metabolic processes. Experimental evidence has demonstrated that freezing of ram sperm compromises mitochondrial physiology by altering the expression of respiratory genes, decreasing ATP content and mitochondrial membrane potential, and elevating ROS levels, ultimately leading to structural mitochondrial damage (Zhang et al. 2024a). Additional studies show that modulation of mitochondrial activity influences key post‐thaw parameters such as membrane integrity, oxidative balance, and metabolic activity (Zarei et al. 2022), while earlier findings indicate that preservation of mitochondrial function correlates with sustained motility and reproductive potential in ovine spermatozoa (Windsor 1997). Cilia and flagella‐associated proteins, Dynein regulatory complex protein 9 and DCC interacting protein 13 alpha, are involved in the architecture and regulation of the sperm flagellum, all of which are enriched in LF rams. Cryopreservation affects the organization and function of the sperm midpiece and principal piece, where central metabolic and motility‐related structures are concentrated. Studies confirm that freezing modifies the abundance and distribution of flagellar‐associated proteins such as hexokinase 1 and casein kinase II alpha, highlighting the sensitivity of the cytoskeleton of rams' sperm to cryoinjury (He et al. 2016). Additional work shows that post‐thaw sperm have altered motility patterns, changes in tyrosine phosphorylation and fluctuations in mitochondrial activity along the flagellum (Peris‐Frau et al. 2020). Since kinematic variables including VSL, VCL, ALH and average path distance depend on the structural integrity and biomechanics of the flagellum, the enrichment of those proteins in LF rams likely reflects molecular disturbances that lower those parameters (Hassan et al. 2024).

Metabolic proteins were also highly abundant in LF rams, particularly Aldo‐keto reductase family 1 member B1, Glycerol kinase, Cytochrome b5 domain‐containing protein 1, Trifunctional enzyme subunit beta, and Adenylate kinase 8. These proteins are linked to energy yield, lipid metabolism, redox regulation, and mitochondrial enzymatic activity, processes known to be impacted by sperm cryopreservation. Freezing disrupts ATP production, alters mitochondrial enzyme function, and contributes to the loss of membrane potential, changes closely related to post‐thaw sperm motility and viability (Zhang et al. 2024a). Experimental interventions targeting oxidative balance or mitochondrial dynamics have been shown to modify membrane integrity, ROS generation, and metabolic performance in ovine sperm (Zarei et al. 2022), emphasizing the importance of metabolic homeostasis during freezing and thawing. Furthermore, cryopreservation affects phosphorylation events and biochemical organization during in vitro capacitation (Peris‐Frau et al. 2020), suggesting that the metabolic proteins predominant in the LF group participate in compensatory responses that arise during freeze–thaw processing. Together, our analyses indicate that reduced cryotolerance in LF rams is associated with disturbances in mitochondrial function, cytoskeletal organization, and metabolic stability.

#### 4.1.3 Clusters of DAPs in Spermatozoa From HF and LF Rams

Proteins with greater abundance in the sperm of HF rams related to pathways of structure and proteases, with focus on protein folding and actin organization. Predominance of chaperones, refolding proteins, and regulators of the cytoskeleton indicated protein stabilization and preservation of cell structure. These mechanisms are fundamental to prevent protein denaturation and cytoskeleton disruption caused by freezing and thawing. In addition, enrichment of post‐translational protein phosphorylation and cell cycle processes indicates the presence of well‐orchestrated protein activity, intracellular signaling, and adaptation to stress in the spermatozoa of rams with superior semen freezability. Enriched clusters of LF ram sperm proteins indicate high dependence on active energy yield and integrity of axonemal structural complexes, making those cells more vulnerable to physical‐chemical alterations caused by cryopreservation. Predominance of catabolic pathways and β‐oxidation of fatty acids indicates high dependence on mitochondrial oxidative phosphorylation for ATP production. This obviously generates more energy for the cell but also accumulates more oxidative stress by‐products, making the sperm cells more susceptible to mitochondrial dysfunction during cryopreservation. Enrichment of fertilization processes appears in the computational biology analysis of sperm proteins of LF rams. This may suggest enhanced fertilizing capacity in vivo, but may also reflect a high propensity for premature capacitation and instability after freezing and thawing. Taken together, these results demonstrate that sperm from LF rams do have normal functional attributes and quality, but with less resistance to cryopreservation in comparison with HF rams. It is important to emphasize that all animals used in the present study had excellent fresh semen quality, and differences among them refer to their responses to cryopreservation, a highly stressful condition. Thus, specific characteristics of their sperm and seminal plasma proteome point toward structural, metabolism and protection mechanisms that make one group (HF rams) better suited for cryoresistance. Proteomic landscapes of LF rams explain why this group responds with lower efficiency to the freezing‐thawing stress, but do not imply that these animals are infertile.

Specific sperm proteins with greater abundance in the LF rams are highly interconnected within specific functional models (glycolysis and pyruvate metabolism, dynein axoneme and molecular motors, mitochondrial respiratory chain). The connection of glycolytic and mitochondrial enzymes obviously indicates that sperm from rams with poor semen freezability experience coordinated oxidative, osmotic, and metabolic stress. As a compensatory mechanism, sperm may upregulate glycolytic and mitochondrial enzymes to maintain energy homeostasis. The increased abundance of these proteins reflects an attempt to counteract energy deficits caused by cryodamage, but this compensation may be insufficient, leading to reduced sperm viability and motility after thawing. The presence of proteins involved in axonemal dynein complexes and flagellar motility indicates that sperm from low‐freezability rams may have altered or damaged flagellar structures. The identification of mitochondrial respiratory chain proteins and EF‐hand domain proteins suggests mitochondrial stress or dysfunction in the sperm of LF rams. These changes may be adaptive responses to cryopreservation stress, but their presence in higher abundance could also indicate underlying vulnerabilities that predispose these sperm to cryodamage and reduced fertility.

#### 4.1.4 Analysis of DAPs in Seminal Plasma From HF and LF Rams

Rams with the HF phenotype had greater abundance of 25 seminal plasma proteins, while other 45 proteins were predominant in LF rams. Molecules associated with protection against oxidative stress, chaperones, epididymal binding proteins, and mitochondrial components were more abundant in the seminal plasma of HF rams. Stress and dismantling/trafficking‐related proteins (such as heat shock 70 kDa protein 13), antioxidants (such as haptoglobin), and mitochondrial subunits (such as cytochrome c oxidase subunit 4 and NADH dehydrogenase subunit 12) were predominant in LF rams. Thus, the overall proteomic profile indicates that HF rams possess a seminal environment that is molecularly more stable, less stress‐responsive, and better equipped to support sperm structural integrity and post‐thaw function when compared with LF rams.

Beta‐defensin had the greatest relative abundance in the seminal plasma of HF rams, as compared to LF rams. This scenario agrees with our previous work, where top‐down proteome analyses identified greater abundance of specific beta‐defensin proteoforms in the seminal plasma of dairy bulls with superior semen freezability (Viana et al. 2025). Studies have shown beta‐defensins associated with increased sperm motility, membrane integrity, and fertilizing capacity in rams and bucks as well (Hall et al. 2017; Solanki et al. 2023). Beta‐defensins are primarily derived from the epididymis and bind to the sperm membrane, playing roles in sperm maturation. These molecules exert antimicrobial activity, modulate immune responses, apoptosis, cell migration, and protection against oxidative stress (Zhu et al. 2020). Rams with the HF phenotype also had higher quantities of WAP four‐disulfide core protein in the seminal plasma, a molecule that contributes to antimicrobial protection and maintenance of membrane integrity (Chen et al. 2024). Thus, beta‐defensin and WAP four‐disulfide core protein contribute to a more stable epididymal and seminal microenvironment favoring overall sperm cryoresistance.

Proteins that act as chaperonins for tubulin and actin, maintaining axonemal/flagellar stability, such as T‐complex protein 1 subunit zeta, were also more abundant in the seminal plasma of rams with high resistance to semen freezing, despite being classified as sperm derived after scrotal insulation disrupted spermatogenesis (Lopez et al. 2015; Rickard et al. 2015). Rickard et al. (2015) and Soleilhavoup et al. (2014) also related enrichment of CCT chaperonins in seminal plasma of rams with superior semen quality, supporting their role in stabilizing cytoskeletal structures critical for post‐thaw motility. Similarly, regulatory proteins involved in cell signaling and protein quality control, such as 14‐3‐3 domain‐containing protein and proteasome subunit alpha type (PSMA3), were identified in high abundance in HF rams provide greater capacity for cell repair and protein refolding (Zimmerman and Sutovsky 2009; Soleilhavoup et al. 2014). The increased abundance of S100 proteins in HF rams is particularly relevant for semen cryopreservation, as these Ca2+‐binding modulators play central roles in preserving membrane integrity, osmotic balance, and regulation of apoptosis during cryogenic stress. Cryopreservation disrupts intracellular Ca2+ homeostasis, triggering premature capacitation and apoptotic pathways. S100 proteins contribute to control Ca2+ fluxes, reducing cryopreservation‐induced membrane destabilization and supporting post‐thaw motility (Soleilhavoup et al. 2014; Zhu et al. 2020). Similarly, the enrichment of ESBP1 in HF rams reflects a more efficient epididymal maturation process. ESBP1 is transferred to spermatozoa via epididymosomes and enhances membrane remodeling, binding capacity, and interaction with the zona pellucida, supporting key functional attributes necessary for fertilization (Leahy et al. 2020). Importantly, because ESPB1 binds exclusively to dead or membrane‐compromised spermatozoa, as demonstrated in bovine (D'Amours et al. 2012), its higher concentration in the plasma seminal fraction of HF rams may indicate that there were fewer dead or damaged spermatozoa available for binding at the time of ejaculation, resulting in a great amount of free ESBP1 detectable in the extracellular fraction. Proteins associated with glycolytic ATP generation, such as phosphopyruvate hydratase (enolase), are also part of the supportive seminal plasma framework for HF. Mitochondrial function is impaired after thawing due to oxidative stress and ice crystal‐induced damage, making glycolysis a critical compensatory pathway to sustain ATP supply to the sperm flagellum. Greater abundance of enolase in HF rams suggests a more robust metabolic capacity to maintain ATP levels under cryogenic stress, supporting vigorous flagellar beating, preventing motility loss, and improving overall post‐thaw recovery (He et al. 2016; Shi et al. 2023). Taken together, the concerted action of Ca2+ regulators (S100), epididymal maturation proteins (ESBP1), and glycolytic enzymes (enolase) represents a molecular profile that directly enhances sperm cryotolerance, explaining the superior post‐thaw motility and membrane integrity observed in HF rams.

The seminal plasma of rams with the LF phenotype had a greater abundance of proteins associated with cellular stress responses, inflammatory responses, and oxidative damage processes, including heat shock proteins (HSPA13), 26S proteasome non‐ATPase regulatory subunit 8 (PSMD8), haptoglobin, and mitochondrial components. Heat shock proteins and proteasome regulatory subunits normally increase when spermatozoa experience thermal or oxidative injury, and act as molecular chaperones assisting the correct folding of proteins and preventing their denaturation (Zhu et al. 2020). Likewise, the lower abundance of PSMD8, which helps degrade damaged proteins and regulate events related to capacitation and membrane remodeling, suggests a more balanced protein environment with controlled protein turnover, without the need to activate the 26S degradative pathway typically triggered by cryo‐induced oxidative lesions. Haptoglobin is related to the cellular response to oxidative stress and tends to increase when there is membrane injury or accumulation of denatured proteins as a compensatory response to cryopreservation‐related damage (Aziz and Ahmad 2013). In addition, the accumulation of mitochondrial proteins such as cytochrome c oxidase subunit 4, involved in ATP generation for flagellar motility (Zhu et al. 2020), and NADH dehydrogenase subunit 12, suggests energetic imbalance and loss of respiratory efficiency, which is closely linked to reduced flagellar motility. Thus, the fact that all these proteins are more abundant in the seminal plasma of LF rams does reflect a functional disruption of the seminal microenvironment, where sperm cells demand greater stress responses (Neila‐Montero et al. 2024). In fact, oxidative, inflammatory, and catabolic protein modules are enriched in the seminal plasma of LF rams, demonstrating a greater requirement for cellular protection and protein quality‐control mechanisms in these animals. This reflects a less stable biochemical environment, with increased oxidative pressure and mitochondrial strain, ultimately compromising membrane integrity, ATP production, and sperm survival during and after freezing. Conversely, the seminal plasma of HF rams is characterized by a more stable microenvironment, with a proteome landscape associated with better antioxidant status and reduced activation of damage‐response pathways, conditions that support superior overall post‐thaw sperm quality.

#### 4.1.5 Clusters of DAPs in Seminal Plasma From HF and LF Rams

The functional pattern of enriched pathways observed in the group of rams with superior semen freezability revealed seminal plasma proteins associated with antigen processing and presentation via MHC class I. Although this pathway is typically linked to immunity, in the seminal context it reflects increased proteasomal activity and a higher capacity for protein turnover, which favors the removal of damaged proteins. This mechanism is crucial during the freezing process, which is known to generate denatured proteins and structural fragmentation. The presence of proteins related to the M phase suggests the involvement of pathways associated with the cytoskeleton and axonemal stability, essential for maintaining sperm motility after thawing. Thus, the HF group demonstrates a profile that supports structural stability, efficient protein quality control, and cellular homeostasis.

Enriched pathways in the group of rams with LF were related to protein folding, cellular responses to stress, and small molecule biosynthetic processes, characterizing cellular stress and compromised protein homeostasis. In the context of sperm cryopreservation, this pattern indicates a basal condition of protein instability, which may limit sperm's ability to maintain functionality during freezing and thawing. In addition, enrichment in cellular responses to stress reinforces the presence of an adverse seminal environment and the activation of adaptive responses to oxidative and metabolic stressors. Activation of the small molecule biosynthetic process suggests compensatory metabolic adjustments, possibly aimed at maintaining energy and redox balance. These pathways reflect a general state of adaptation to damage rather than functional efficiency, which is consistent with the lower seminal quality and reduced resistance to cryopreservation observed in the LF group.

This study shows how the intricate nature of sperm and seminal plasma proteomes potentially defines sperm cryoresilience. This knowledge will support initiatives for the development of new protocols for semen cryopreservation and sire selection.

## 5 Conclusion

The present study demonstrates that rams with HF and LF exhibit distinct proteomic profiles in spermatozoa and seminal plasma from fresh semen. The sperm proteome of HF rams was enriched with extracellular and immune‐related proteins, antiproteases, chaperones, and structural regulators, supporting cytoskeletal integrity, apoptotic resistance, and proteostasis. In contrast, mitochondrial subunits, axonemal components, and bioenergetic enzymes were predominant in the sperm proteome of LF rams, indicating greater reliance on oxidative metabolism. The seminal plasma of HF rams exhibited proteins mainly associated with membrane stabilization, calcium regulation, epididymal maturation, and ATP availability, whereas the seminal fluid of LF rams was characterized by proteins linked to cellular stress, inflammatory responses, and oxidative damage. These findings establish a molecular framework underlying individual variation in frozen‐thawed semen parameters in tropically adapted rams, providing a basis for the identification of potential biomarkers of semen cryoresistance and improvement of methods for semen cryopreservation and sire selection.

## Author Contributions

**Wallisson Bruno de Morais Pacheco:** conceptualization, investigation, data curation, writing – original draft, writing – review and editing, formal analysis, methodology, visualization, validation. **Iran Aguila Maciel:** conceptualization, investigation, writing – original draft, writing – review and editing, formal analysis, data curation, methodology, visualization, validation. **Suane Santos Alves dos Santos:** validation, investigation. **Alan Martins de Araújo:** investigation. **Francisca Nágila Matos Almeida:** investigation. **Denise Damasceno Guerreiro:** methodology, validation, investigation, formal analysis, writing – review and editing. **Sheheryar Sheheryar:** methodology, validation, investigation, formal analysis. **Lais Lacerda Brasil de Oliveira:** resources. **Felipe Domingos Sousa:** resources. **Mohibullah Shah:** writing – review and editing, validation. **Maria Elisabete Amaral Moraes:** resources. **Carlos Roberto Koscky Paier:** resources. **Ana Cristina de Oliveira Monteiro:** resources. **Carminda Sandra Brito Salmito‐Vanderley:** funding acquisition, validation. **Arlindo A. Moura:** supervision, conceptualization, writing – review and editing, funding acquisition, resources, formal analysis, project administration.

## Ethics Statement

All animal procedures were ethically reviewed and approved by the Comissão de Ética para o Uso de Animais (CEUA) of the Universidade Estadual do Ceará (UECE), under protocol number 10420061/2022. All procedures complied with institutional and national guidelines for the care and use of laboratory animals.

## Conflicts of Interest

The authors declare no conflicts of interest.

## Supporting information


Supporting File 1

Supporting File 2

## Data Availability

The mass spectrometry proteomics data have been deposited in the ProteomeXchange Consortium via the PRIDE partner repository with the dataset identifier PXD068437 and DOI 10.6019/PXD068437. During peer review, the dataset can be accessed using the following reviewer credentials: Project accession PXD068437, Token sPDicb2wX43K, or via the reviewer account (username: reviewer_pxd068437@ebi.ac.uk; password: 6WQPdvO3nMas). After publication, the dataset will become publicly accessible without the need for login credentials.
